# Contrasting roles of APE1 and APE2 in genome maintenance, cancer development, and therapeutic targeting

**DOI:** 10.1093/narcan/zcaf048

**Published:** 2025-12-23

**Authors:** Aman Sharma, Helen E Grimsley, Katharine Courtemanche, Simon N Powell

**Affiliations:** Department of Radiation Oncology, Memorial Sloan Kettering Cancer Center, NY, NY 10065, United States; Department of Radiation Oncology, Memorial Sloan Kettering Cancer Center, NY, NY 10065, United States; Department of Radiation Oncology, Memorial Sloan Kettering Cancer Center, NY, NY 10065, United States; Department of Radiation Oncology, Memorial Sloan Kettering Cancer Center, NY, NY 10065, United States

## Abstract

Apurinic/apyrimidinic endonucleases - APE1 and APE2 are central to genome maintenance and the cellular DNA damage response, with expanding relevance in cancer biology. APE1 is the primary endonuclease in base excision repair and functions as a redox coactivator of transcription factors. In contrast, APE2 exhibits PCNA dependent 3′–5′ exonuclease and 3′-phosphodiesterase activities, contributing to microhomology-mediated end joining, ATR-Chk1 activation, and immunoglobulin diversification. Both enzymes are often deregulated in cancer: APE1 is frequently overexpressed, drives tumor progression and chemoresistance, while APE2 is similarly upregulated in multiple malignancies. APE1 can be targeted by redox-specific or endonuclease inhibitors, with early clinical evidence of biological activity and tolerability. Although APE2-specific inhibitors remain in early development, emerging synthetic lethality data and preclinical studies highlight APE2 as a novel clinical target in breast cancer type 1/2 susceptibility (BRCA)-mutated cancers. This review discusses the structural and functional roles of APE1 and APE2, their contributions to cancer biology and therapeutics, recent advances in inhibitor development, and future strategies for precision oncology.

## Graphical Abstract

**Graphical Abstract:**
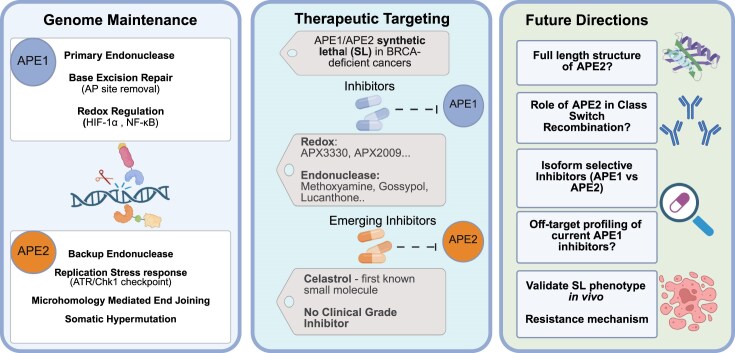

## Introduction

Genomic integrity is continuously challenged by endogenous (e.g. reactive oxygen species, DNA secondary structures) and exogenous (e.g. ultraviolet light, ionizing irradiation) agents that cause DNA damage. Among these DNA lesions, apurinic/apyrimidinic (AP) sites are particularly abundant (∼10 000 per cell per day) due to spontaneous base loss, oxidative damage, and enzymatic removal by lesion-specific DNA glycosylases [1]. If left unrepaired, these lesions compromise genome stability, underscoring the need for efficient repair mechanisms.

Mammalian cells rely on several DNA repair pathways, including nucleotide excision repair, base excision repair (BER), mismatch repair, homologous recombination (HR), Alterative end joining, and nonhomologous end-joining (NHEJ) [2]. Among these, BER primarily fixes small base lesions that generates AP sites [3]. In BER, a damaged base is removed by a DNA glycosylase, leaving an AP site that is incised by an AP endonuclease; the resulting gap is then filled by DNA polymerase and sealed by DNA ligase. The AP endonucleases (APE1 and APE2) are the major endonuclease in mammals that initiates the repair at abasic sites.

APE1 and APE2 are closely related paralogs in the exonuclease III (ExoIII) family with substantial sequence and structural homology but distinct functions [4–6]. APE1 (APEX1) is the dominant AP endonuclease, responsible for >95% of AP site repair, and uniquely serves as a redox co-regulator of transcription factors (TFs). APE2 (APEX2), by contrast, has minimal AP incision activity but exhibits 3′–5′ exonuclease activity and is specialized for replication stress responses via its PCNA-interaction. [7, 8]. APE1 is ubiquitously expressed and is essential for viability—loss of the APEX1 gene is embryonic lethal in mice [9], underscoring its critical role as the primary AP endonuclease. Both enzymes contribute to ATR (Ataxia telangiectasia and Rad3 related)–Chk1 checkpoint activation under replication stress [10, 11].

Both APE1 and APE2 are implicated in cancer biology. APE1 overexpression is common across tumor types, correlating with genomic instability, therapy resistance, and poor prognosis [12]. APE2 is also dysregulated in cancers and overexpression patterns linked to replication stress survival in BRCA-deficient cancers [13]. Recent studies show that inhibiting APE1 or APE2 can be synthetic-lethal in HR-defective cancers, highlighting them as emerging therapeutic targets [14–16]. Despite these advances, important questions remain. The full-length structure of APE2 as well as the disordered N-terminal tail of APE1 has not yet been resolved, isoform-specific inhibitors for APE1 versus APE2 are lacking, and their interplay in specialized processes such as immunoglobulin diversification remains incompletely defined. This review examines the structural and functional roles of APE1 and APE2, their contributions to genome maintenance and cancer etiology, and recent advances in therapeutic strategies aimed at exploiting their vulnerabilities.

## Structural features and localization

APE1 and APE2 are the major AP endonucleases in humans and members of the Exo III family. Both enzymes share the conserved exonuclease/endonuclease/phosphatase (EEP) fold and similar catalytic residues to coordinate a divalent metal ion. APE2 carries a unique C-terminal extension with a PCNA-interacting protein (PIP) motif and a zinc-finger (Zf-GRF) domain that targets APE2 to replication forks for replication-associated DNA repair [17].

### APE1

**Figure 1.**
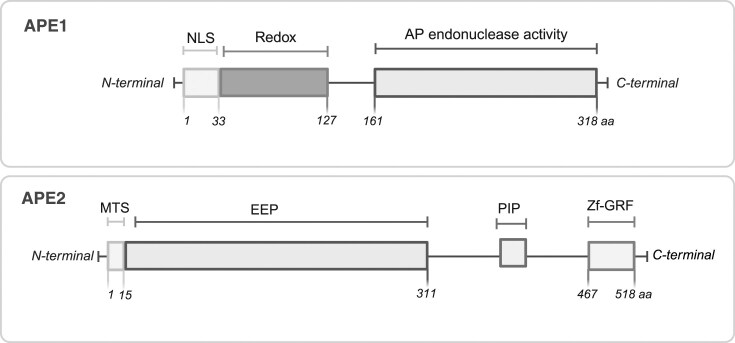
Structural domains of the human APE1 (top) and APE2 (bottom).EEP, exonuclease/endonuclease/phosphatase; MTS, mitochondrial targeting signal; NLS, nuclear localization signal; PIP, PCNA-interacting protein. Created in BioRender. Grimsley, H. (2025; https://BioRender.com/l9ar51o).

The human APE1 is a well-characterized ∼35 kDa protein with distinct functional regions for its dual activities—DNA repair and redox regulation (Fig. 1). The N-terminal tail (∼1–42 amino acids) is intrinsically disordered and is responsible for the redox activity of APE1. This flexibility is suggested to facilitate its interactions with various TFs, allowing APE1 to influence multiple signaling pathways [18]. The tail harbors multiple lysine (Lys) residues that are acetylated (Lys27, Lys31/32, and Lys35) and linked to RNA metabolism [19]. Under genotoxic stress, APE1 is acetylated at Lys6/7 residues to enhance BER and the mechanism is antagonized by sirtuin-1 (SIRT1) deacetylase [20]. APE1 also contains key cysteines that undergo reversible oxidation-reduction, enabling its redox sensor role [21]. The C-terminal domain of APE1 adopts the conserved EEP fold that carries the AP–endonuclease active site [22] (Fig. 1). This domain coordinates a metal ion, typically Mg²⁺ or sometimes Mn²⁺, which helps to activate water molecules that attack the phosphodiester bond adjacent to the AP sites, resulting in a single-strand DNA break (SSB) with a 3′-hydroxyl and a 5′-deoxyribose phosphate end [23]. Structural studies show that APE1 binds DNA by bending the helix and flipping an AP site out of the duplex and into its active-site pocket [23]. Consistent with these earlier findings, a recent cryogenic electron microscopy (Cryo-EM) structure of full–length APE1 bound to an AP site-containing nucleosome core particle (APE1–AP-NCP complex at ∼3.4 Å) demonstrates that APE1 bends the DNA and flips the AP site out of the DNA helix into its active site by a DNA sculpting mechanism [24].

### Cellular localization of APE1

APE1 is predominantly localized in the nucleus but can localize in mitochondria under certain conditions [25]. The nuclear localization is mediated by the nuclear localization sequence (NLS) sequence located within the first 33 amino acids (Fig. 1). Deletion of these residues causes APE1 to mislocalize to the cytoplasm [26]. The nuclear import of APE1 is facilitated by importin proteins, which recognizes the NLS [26]. APE1 displays a dynamic nuclear–cytoplasmic distribution, which is regulated in response to cellular stress, particularly oxidative stress and DNA damage. Under unstressed conditions, APE1 is abundant in the nucleus, but subsets translocate to the mitochondria, nucleolus, or cytoplasm which modulate its recruitment to distinct pathways. APE1 contains an unconventional mitochondrial targeting sequence (MTS) at residues 289–318, enabling a role in mitochondrial BER [27]. The mitochondrial APE1 is present at low basal levels than its nuclear counterpart but increases when mitochondrial oxidative DNA damage is elevated [28]. APE1 can also be sequestered in the nucleolus via interaction with nucleophosmin (NPM) [29]. These localization mechanisms ensure that engaging APE1 in one pathway does not deplete the enzyme for its other functions.

### APE2

APE2 is a ∼60 kDa protein composed of an N-terminal (EEP) catalytic domain, a C-terminal zinc finger domain with a conserved GRxF residues (termed Zf-GRF) and a flexible region that includes a PIP motif (∼390–397 amino acids) in between the C- and N-termini (Fig. 1) [8]. The first ∼15 amino acid sequence of the N-terminal domain possesses an MTS [30], suggesting its role in mitochondria BER as well. X-ray studies of the Zf-GRF domain (residues 445–517) from Xenopus APE2 show it forms a flexible, claw-like shape attached to the catalytic core. This allows APE2 to bind single-stranded DNA (ssDNA) and process complex 3′ DNA lesions, coordinating ATR–Chk1 signaling via RPA [31]. The unique Zf-GRF domain positions APE2 as a vital regulator of genome stability in chromatin inaccessible to APE1. At present, no full-length APE2 structure is available, but some evidence establishes its domain layout and structural interaction with PCNA [31, 32].

### Cellular localization of APE2

APE2 has dual localization capacity, with an N-terminal MTS and nuclear localization via interaction with PCNA. The MTS drives APE2 localization into mitochondria, as shown by electron microscopy and immunostaining via GFP fusion study [30]. APE2 lacks a classic NLS; instead, its PIP motif mediates nuclear localization by binding PCNA during DNA replication/repair [30, 32]. Under oxidative stress, APE2 forms PCNA-containing nuclear foci, and PCNA binding greatly stimulates APE2’s exonuclease activity [32], highlighting its role in PCNA-dependent repair of oxidative DNA damage. Thus, APE2’s localization is governed by its intrinsic signals: the N-terminal MTS for import into mitochondria, and the PIP/PCNA-binding motif for retention at nuclear DNA damage sites. This regulated mobilization of APE2, dictated by protein–protein interactions and DNA context, helps prevent functional conflict with APE1’s functions, and allows the two enzymes to either complement each other or even take on opposing roles in different pathways. Despite the detailed structural knowledge of APE1, the complete structure of APE2 remains unresolved [33]. This gap impedes understanding of how APE2 coordinates its domains during DNA repair when bound to PCNA. Furthermore, it’s not well understood how APE2’s domain-specific activities are regulated in cells—by protein partners or post-translational modifications.

## Enzymatic activities of APE1 and APE2

### AP endonuclease activity

APE1 exhibits robust endonuclease activity (with a catalytic efficiency of 100 s^–1^ μM^–1^) [34, 35], cleaving AP sites at the rate of ∼700 per second [35]. The abasic sugar is flipped out of the helix into the APE1’s active site pocket [36, 37]. Cryo-EM studies show a ‘base sculpting’ mechanism: APE1 bends the DNA and flips out the AP site to facilitate BER processing [24]. XRCC1 stabilizes the APE1–DNA complex, enhancing APE1’s processivity [38].

In cells, APE1 incises >95% of AP site, leaving APE2 as only a minor contributor [39, 40]. *In vitro* assays using a double-stranded DNA substrate containing a single AP site show that wild-type human APE2 generates only a small fraction (∼7%) of incision product (e.g. using a tetrahydrofuran AP-analog in a 75-nt duplex), whereas APE1 cleaves it efficiently [39]. Structurally, APE2’s active site lacks the hydrophobic pocket that makes APE1 so efficient at AP cleavage; inserting those missing residues into APE2 significantly boosts its AP endonuclease activity [41]. Thus, APE2 processes abasic sites only as a low-efficiency backup in BER.

### 3′–5′ exonuclease activity

APE1 also has an exonuclease activity (with a catalytic efficiency of 1 s^–1^ μM^–1^) that excises mismatched or damaged 3′-termini from DNA nicks [34, 42]. APE1’s exonuclease is active at both matched (kcat of 2.3 min⁻¹) and mismatched 3′ termini (kcat of 61.2 min⁻¹), with a clear preference for mismatched bases [43, 44]. The exonuclease activity of APE1 is slower than its endonuclease function (>100-fold difference), but either faster or within a single order of magnitude (up to 10-fold higher or lower) of end-processing enzymes such as APE2, tyrosyl-DNA phosphodiesterase 1 (TDP1), and polynucleotide kinase (PNK) [34]. Thus, this exonuclease activity of APE1 is likely to be biologically relevant, although its precise physiological role awaits confirmation (e.g. through genetic or inhibitor studies). Recent crystal structures reveal how APE1’s single active site accommodates both AP-endonuclease and 3′–5′ exonuclease activities. Freudenthal *et al.* (2015) solved APE1 bound to product DNA defining the metal ion coordination, nucleophile position, and key arginine clamps that facilitate catalysis [45]. Likewise, Whitaker *et al.* (2018) determined multiple APE1-DNA complex structures showing how the single active site accommodates and removes diverse 3′-mismatches and DNA damage [46]. These structures reveal that APE1 bends the DNA and slots a 3′-mismatched or damaged base into an intra-helical pocket, enabling efficient excision without base-flipping.

*In vitro* studies utilizing recombinant human APE2 show robust 3′–5′ exonuclease activity on duplex DNA [39]. It preferentially trims 3′-mismatched or 3′-terminal nucleotides located at nicked, blunt-ended, or recessed DNA substrates [39]. Binding to PCNA via its N-terminal PIP motif greatly stimulates APE2’s exonuclease processivity, significantly enhancing 3′ resection and removal of blocked ends [47]. Like APE1, APE2 uses a metal-coordinated water for catalysis (with Mn²⁺ supporting higher activity than Mg²⁺) [39]. By excising a few nucleotides from the 3′ end, APE2 extends the ssDNA gap, which facilitates ATR checkpoint activation [48].

### 3′-Phosphodiesterase activity

APE1 can remove certain 3′-blocking groups (e.g. 3′-phosphate or 3′-phosphoglycolate), albeit lower efficiency than dedicated enzymes such as PNKP or TDP1 [49, 50]. Biochemical fractionation and immunodepletion studies showed that APE1 accounts for the majority of 3′-phosphoglycolate end-processing activity in human cell extracts [51]. On the other hand, APE2 processes blocked 3′ termini (from oxidative damage, a misincorporated ribonucleotide, or an abortive topoisomerase event). Although intrinsically weak, this activity is markedly enhanced by PCNA binding, enabling APE2 to complement other repair factors in resolving complex 3′ends. [16, 32, 39, 52]. APE2 trims the peptide-linked 3′ ends of TOP1 cleavage complexes (Top1cc), complementing TDP1’s activity in resolving these lesions [53].

### Endoribonuclease activity

APE1 can incise oxidized ribonucleotides embedded in DNA about as efficiently as it processes canonical AP sites [54]. APE1 also cleaves RNA in a manner like RNase H, targeting specific pyrimidine–purine sites; modulating the stability of select transcripts such as c-myc [55–57]. This function links APE1’s redox and repair activities to post-transcriptional regulation. Unlike APE1, APE2 has not been shown to cleave RNA.

Despite these differences, both enzymes share the conserved Exo III-family EEP catalytic core, using acidic residues and metal ion coordination for phosphodiester bond cleavage. However, direct comparative kinetic analyses remain limited. Future studies under physiological conditions are needed to clarify their overlapping versus specialized functions and to identify which enzymatic activities might be selectively targeted for therapeutic benefit.

## Biological functions

### Roles of APE1/Ref-1

#### Central player in DNA repair

**Figure 2.**
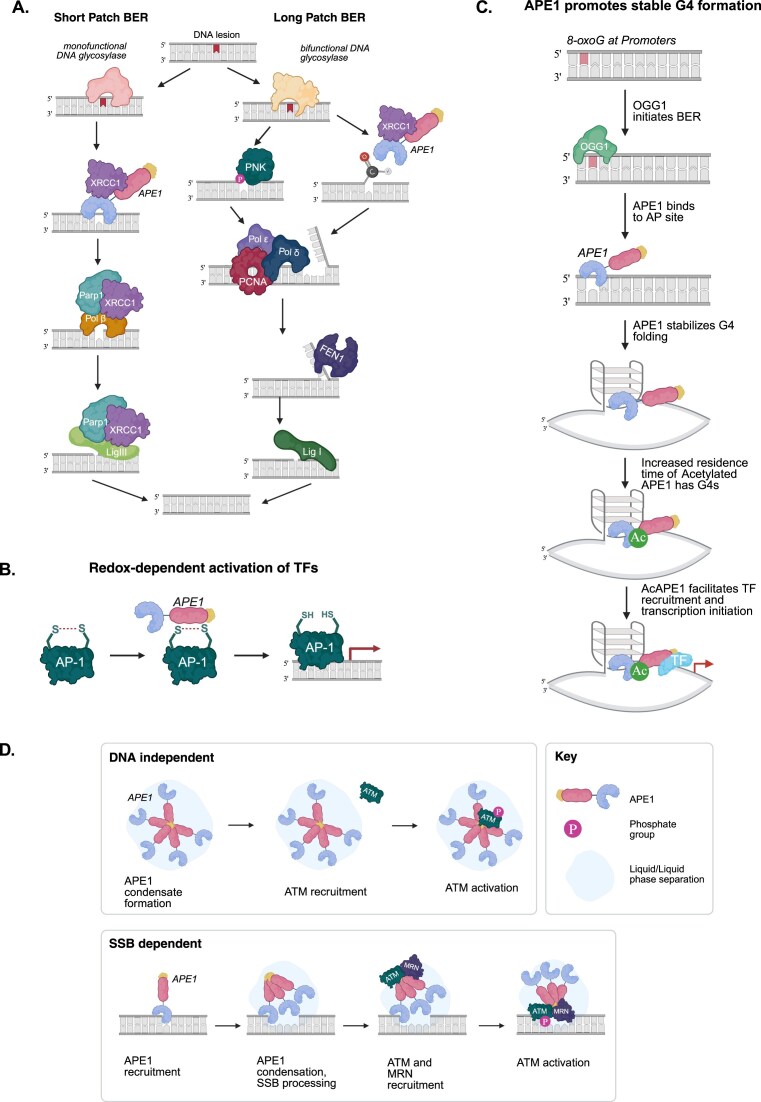
Biological functions of APE1. (**A**) Schematic diagram of (left) short patch and (right) long patch BER pathway. (**B**) APE1 reduces cysteine residues in TFs such as AP-1 to restore their DNA-binding activity and facilitating transcription. (**C**) Schematic diagram of oxidized base damage (8-oxoG), AP site formation by OGG1, and binding of APE1 to the AP sites at promoter quadruplex site promotes the formation and stabilization of a G-quadruplex (G4) structure. Acetylation of APE1 by p300 increases residence time of APE1 on G4 structures which stimulates loading of TFs. (**D**) Two distinct mechanisms of APE1 mediated SSB-induced Ataxia-telangiectasia mutated (ATM) signaling—DNA-independent model and SSB dependent model. Created in BioRender. Grimsley, H. (2025; https://BioRender.com/8c707ii).

APE1 is the central endonuclease in BER, which repairs abasic sites generated by DNA glycosylases from nonbulky lesions such as oxidized or alkylated base, deamination, and spontaneous base loss. This rapid phenomenon does not require a stable glycosylase–APE1 complex [58]. APE1 cleaves the phosphodiester backbone at AP sites, creating a nick with a 3′-hydroxyl group and a 5′-deoxyribose phosphate terminus. This nick is essential for the subsequent actions of DNA polymerase β (Polβ) to fill the gap, and DNA ligase to seal it [3]. The role of APE1 extends beyond its enzymatic function as it interacts with other proteins involved in BER, such as XRCC1, enhancing the efficiency and coordination of the BER pathway [38]. The BER pathway comprises two branches: short- and long-patch BER (Fig. 2A). In short-patch BER (single-nucleotide replacement), Polβ removes the 5′-dRP (via its lyase activity) and fills the gap, and XRCC1-LigIIIα ligates the nick. On the other hand, long-patch BER (2–10 nt) involves replicative Polδ/ε polymerases (with PCNA) that displaces a short flap which is cleaved by Flap endonuclease (FEN1), followed by ligation by Ligase I [59].

APE1 can also initiate nucleotide incision repair (NIR) by directly cleaving certain oxidized bases (e.g. 5-hydroxyuracil) without a glycosylase [60]. This involves both the endonuclease and exonuclease activities of APE1. APE1 removes 3′-blocking groups at DNA strand breaks (such as 3′-phosphate or 3′-phosphoglycolate residues) to generate repairable 3′-hydroxyl ends, and it can directly incise certain oxidized or damaged bases via NIR [61]. High-resolution crystallography has revealed the intricate details of APE1’s active site and its interaction with damaged DNA, enhancing our understanding of its substrate specificity and catalytic mechanism [45]. APE1 may even assist in repairing double-strand breaks (DSBs) or interacting with other repair pathways such as NHEJ, though these functions are less understood [62, 63].

#### Redox signaling

Beyond DNA repair, APE1 functions as a redox signaling factor (Ref-1) that modulates various TFs through redox-dependent mechanisms. This redox activity relies on critical cysteine residues in APE1 [64, 65]. APE1 reduces oxidized cysteines in TFs via a thioredoxin-dependent thiol exchange mechanism, restoring their DNA-binding activity and facilitates the transcription of genes involved in oxidative stress responses (Fig. 2B) [66]. By modulating the redox state of these TFs, APE1 affects their ability to bind DNA and regulate gene expression, thereby impacting various physiological and pathological processes. APE1’s redox regulation extends to several key TFs, including AP-1, NF-κB, HIF-1α, and p53 [67–70]. The redox function of APE1 is tightly regulated by post-translational modifications and protein interactions. For example, phosphorylation of APE1 by casein kinase II enhances its redox activity, while interaction with NPM regulates its subcellular localization and function [71, 72]. APE1 maintains p53 in its reduced state by targeting its DNA-binding domain, which is crucial for its ability to activate target genes involved in cell cycle arrest and apoptosis [73–75].

#### Transcriptional regulation via G4 binding

APE1 also influences gene expression via binding to secondary structures such as G4 structures [76]. G4s are formed when four guanine bases engage in Hoogsteen hydrogen bonding, creating a G-quartet which self-stack to generate a quadruplex. Genome-wide studies have identified G4-rich regions at gene promoters/enhancers, replication origins, and telomeres [77]. These structures are prone to oxidative damage and often require BER at those sites [78]. Many oncogenes (e.g. MYC, KRAS) have promoter G4 sequences that regulate their expression [79]. APE1 binding to oxidized G4-rich promoter region promotes G4 folding and facilitates TFs loading (Fig. 2C). Mechanistically, oxidative base damage in G4-forming promoter regions yields an AP site; APE1 binds this site, promoting G4 formation. APE1 is acetylated by p300, which prolongs its chromatin association [80]. The acetylated APE1–G4 complex recruits transcription machinery to initiate transcription: for instance, APE1–G4-mediated activation has been observed at KRAS, c-MYC, and BCL-2 [81–83]. Recently, APE1 was shown to regulate urea cycle metabolism via promoter G4 structures in the CPS1 and ARG2 genes [84]. Overall, APE1’s redox and G4 function links the oxidative stress to transcriptional control of oncogenes and stress-response genes.

#### RNA processing

APE1 also functions as an endoribonuclease that cleaves abasic or oxidized RNA [29, 85]. Studies have shown that APE1 can influence the stability of specific messenger RNAs (mRNAs), either by directly cleaving them or by interacting with RNA-binding proteins that regulate mRNA decay. For example, APE1 cleaves *c-myc* mRNA, contributing to its degradation and downregulation of c-Myc protein levels [56]. APE1 can even localize to nucleoli and bind ribosomal RNA (rRNA; e.g. G4 RNA), suggesting a role in rRNA quality control under oxidative stress [86]. APE1 has been implicated in microRNAs (miRNAs) biogenesis; it can cleave or remodel specific precursor miRNAs [87]. For instance, APE1 binding to a G4 motif in pre-miR-92b influences that miRNA’s maturation and localization [88]. Cells lacking APE1 accumulate unprocessed pri-miRNAs and show misregulation of corresponding targets, highlighting APE1’s broader role in RNA metabolism.

#### Telomere maintenance

Telomeres are G-rich regions and susceptible to oxidative damage, APE1 helps repair lesions (such as 8-oxoG bases removed by OGG1) to prevent telomere instability [89]. Dysfunctional telomeres can trigger chromosomal end-to-end fusions, leading to genomic instability and potentially cancer [90]. APE1 plays an essential role in maintaining telomere stability by physically interacting with the shelterin complex proteins TRF1 and TRF2, and POT1 [91–94]. APE1 depletion causes TRF2 to dissociate from telomeres, resulting in shortening/uncapping, fusion events and DNA damage [93]. By supporting TRF2 function and reinforcing the shelterin complex, APE1 helps preserve telomere protection and length regulation.

#### ATM DNA damage response pathway activation

The ATM-mediated DNA damage response (DDR) pathway is generally activated in response to DSBs or oxidative stress [95]. Recently, Zhao *et l.* (2024) used a plasmid-based SSB structure in Xenopus egg extracts, suggesting that APE1 performs activation of ATM-dependent DDR signaling through both enzymatic and scaffolding mechanisms [96]. The study identified two distinct modes by which APE1 activates ATM—one requiring DNA (SSB-dependent) and one even in the absence of DNA (DNA-independent) (Fig. 2D) [11].

##### SSB-dependent model

APE1 uses its 3′–5′ exonuclease activity to resect the DNA ends at an SSB, converting the SSB into a short ssDNA gap. This resection generates an intermediate resembling an early DSB, thereby promoting ATM kinase recruitment. After resection, APE1 oligomerizes into molecular condensates that recruit ATM and the MRE11–RAD50–NBS1 (MRN) complex, leading to direct ATM activation. Interestingly, depletion of APE1 (but not APE2) impairs SSB-induced ATM signaling [96].

##### DNA independent model

APE1 protein forms dimers or oligomers when overexpressed in Xenopus egg extracts. These APE1 oligomers form condensates via its N-terminal motif, in the absence of DNA. These condensates recruit ATM kinase, leading to its direct activation by APE1 [96].

The precise mechanisms leading to APE1-driven condensate assembly and disassembly remain to be elucidated. Future studies employing advanced biophysical and single-molecule approaches, such as mass photometry, super-resolution imaging, will provide a detailed mechanistic insight.

### Roles of APE2

#### DNA repair mechanisms

**Figure 3.**
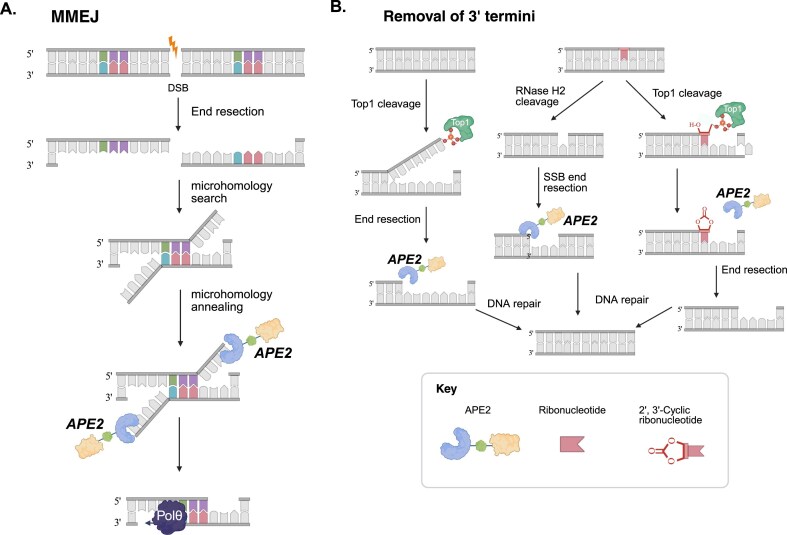
Biological functions of APE2. (**A**) Schematic of the MMEJ repair pathway. The exonuclease activity of APE2 removes 3′-Flaps structures generated during MMEJ (**B**) Schematic diagram of the 3′–5′ exonuclease activity of APE2 is required to perform resection at an SSB, removing 3′-blocking ends and TOP1 cleavage complexes, which facilitates SSB repair and gap filling. Created in BioRender. Grimsley, H. (2025; https://BioRender.com/bi077f8).

APE2 participates in multiple DNA repair mechanisms, such as microhomology-mediated end joining (MMEJ), and SSB repair. MMEJ is an error-prone backup pathway, which involves repair proteins such as Poly (ADP-ribose) polymerase 1 (PARP1), DNA polymerase theta (Polθ), Flap Endonuclease 1 (FEN1), and DNA Ligase III (LIG3). MMEJ utilizes short regions of microhomology (∼3 to 20 nucleotides) for rejoining [97]. Loss of APE2 impairs MMEJ activity, preventing efficient fusion of broken DNA ends at deprotected telomeres and intra-chromosomal DSBs [98]. Interestingly, deleting APE2’s C-terminal Zf-GRF zinc-finger (but not the PCNA-binding PIP motif) abolishes its ability to rescue MMEJ, indicating APE2’s role in MMEJ is PCNA-independent. Mechanistically, APE2 has a 3′ flap endonuclease activity that helps remove overhangs at microhomologous ends, and its unique C-terminal domain recruit APE2 to DSB sites to facilitate end-joining (Fig. 3A) [47].

APE2 also contributes to SSB repair, especially under conditions of oxidative stress (e.g. H_2_O_2_ exposure). APE2 can process AP sites and exhibits 3′–5′ exonuclease and 3′-phosphodiesterase activities, which are greatly enhanced by PCNA interaction [32, 99]. The exonuclease activity of APE2 can resect DNA at an SSB, removing 3′-blocking ends or short oligonucleotides, which facilitates SSB repair and gap filling (Fig. 3B) [31]. Thus, during oxidative stress when many SSBs with damaged ends accumulate, APE2 becomes particularly crucial. Notably, APE2 localizes to mitochondria also suggests it helps repair oxidative DNA damage in mitochondrial genomes [30].

#### DDR signaling

APE2 plays a role in DDR signaling pathways, especially in the activation of checkpoint pathways such as p53 tumor suppressor pathway. APE2 deficiency leads to accumulation of unresolved DNA breaks and replication stress, which can trigger checkpoint activation. In germinal center B cells, APE2-deficient cells accumulate excess DSBs during rapid proliferation, resulting in a p53-independent G₁/S arrest followed by p53-dependent apoptosis [100]. This indicates that APE2 helps resolve DNA lesions in proliferating cells, preventing chronic checkpoint activation. In p53-proficient cells, loss of APE2 is sensed as excessive genomic stress, stabilizing p53, cell-cycle blockade, and apoptosis. Conversely, in p53-deficient backgrounds, cells lacking APE2 might tolerate more genomic chaos. Thus, APE2 collaborates with the p53 pathway to limit DNA damage that would otherwise hyperactivate p53. How APE2 influences p53-dependent cell-cycle checkpoints is not completely clear. Recent studies have shown that APE2 has also been linked to regulating the ATR–Chk1 checkpoint pathway [101].

### Coordinated role of APE1 and APE2

#### Immune system functions

Class switch recombination (CSR) and somatic hypermutation (SHM) are initiated by activation-induced deaminase (AID), which deaminates cytosine to uracil, generating U:G mismatches [102]. These lesions are processed by BER and mismatch repair to create DSBs (CSR) or point mutations (SHM). Both APE1 and APE2 contribute to antibody diversification, though their roles differ and remain debated [102].

APE2 is highly upregulated in germinal center B cells and promotes the mutagenic repair of AID-induced lesions, whereas APE1 is downregulated in this context [103, 104]. In SHM, APE2 activity at AP sites drives error-prone repair: incision of an AP site in the DNA coding strand creates a single-strand breaks that is processed into mutations or small indels at A:T base pairs. After UNG removes uracil, APE2 incises the AP site and, via PCNA binding, uses its exonuclease/phosphodiesterase activity to trim the DNA end. Monoubiquitinated PCNA then recruits translesion polymerases (Pol η, Rev1) to fill the gap, introducing mutations [105, 106]. In parallel, APE2-generated nicks can trigger long-patch excision by MSH2–MSH6–EXO1, creating larger gaps that TLS polymerases fill (the source of most A:T mutations). APE2-knockout mice display markedly reduced SHM, especially loss of A:T mutations, confirming its nonredundant role [103]. Recent work suggests that DNA repair factor HMCES protects AP sites on ssDNA by forming a covalent crosslink with the AP site, which suppresses APE2 cleavage activity to prevent unwanted DSBs during SHM [107]. Thus, APE2 is a central driver of mutagenic SHM, critical for antibody diversity.

In contrast to its limited role in SHM, APE1 is implicated as the critical nuclease for CSR [108]. Deleting APE1 nearly abolishes CSR, whereas deleting APE2 alone shows variable effects [108]. Evidence suggests APE1’s main role is processing AID-induced switch-region breaks rather than initiating them [109]. Evidence from primary splenic B cells supports contributions in CSR from both APE1 and APE2 [110]. Guikema *et al.* (2007) showed that B cells deficient in APE1, APE2, or both had reduced CSR to multiple isotypes and fewer detectable S-region DSBs [110]. In splenic B cells isolated from APE2-deficient mice, CSR was reduced to ∼65% relative to wild-type cells, indicating that APE2 contributes to the efficiency of CSR, certain IgG isotypes were especially affected, while IgA remained intact [110]. This suggests that APE2 may help generate or resolve staggered DSBs during CSR. However, other groups saw little to no CSR defect in APE2-null cells (varying by strain and isotype), underscoring that APE2’s role is context-dependent [111]. APE2-null B cells accumulate unrepaired switch-region breaks and trigger p53-dependent and independent checkpoints, implying that without APE2 the balance shifts from productive CSR to a DDR [100].

The precise contribution of APE2 to CSR remains debated, with some studies finding it necessary for generating switch-region breaks and others finding it largely dispensable. These discrepancies may reflect in differences in model systems, genetic backgrounds, or compensatory repair pathways. One proposed model is that APE1 and APE2 have complementary functions: APE1 ensures end cleavage and high-fidelity repair, while APE2 promotes error-prone processing (SHM) and can enhance DSB formation in CSR under certain conditions. It remains unclear whether APE2’s role is context-dependent or if compensatory pathways mask its importance. Together, these findings highlight the nonredundant but context-dependent functions of APE1 and APE2 in antibody diversification.

Polymorphisms in the APE1 and APE2 genes can alter immune function and potentially affect cancer risk. APE1 polymorphisms have been linked to immune and inflammatory diseases by affecting DNA repair efficiency, cytokine production, and redox signaling [18]. The most widely studied APE1 polymorphism is D148E variant [112, 113], associated with various cancers and with infectious diseases like meningitis [114–119]. The study on bacterial meningitis patients carrying the D148E variant showed lower IL-6/IL-8 levels and a higher IgG:IgA ratio [117]. This suggests a reduced inflammatory response and altered antibody production. However, APE2 polymorphisms remain poorly studied and warrant further investigation. Given that APE2 plays immune roles, variants in APE2 could impair adaptive immunity and thus impact tumor surveillance, warranting further investigation.

#### Interplay of APE1 and APE2 in ATR/ATRIP checkpoint activation

ATR kinase is a critical replication-stress checkpoint activated by stalled forks [120]. The ATR–ATRIP binds RPA-coated ssDNA and, with the 9–1–1 clamp and TopBP1, triggers CHK1 to stabilize the fork [121]. Studies in Xenopus extracts and mammalian cells reveal that APE1 and APE2 act sequentially to generate the ssDNA required for the ATR activation pathway.

**Figure 4.**
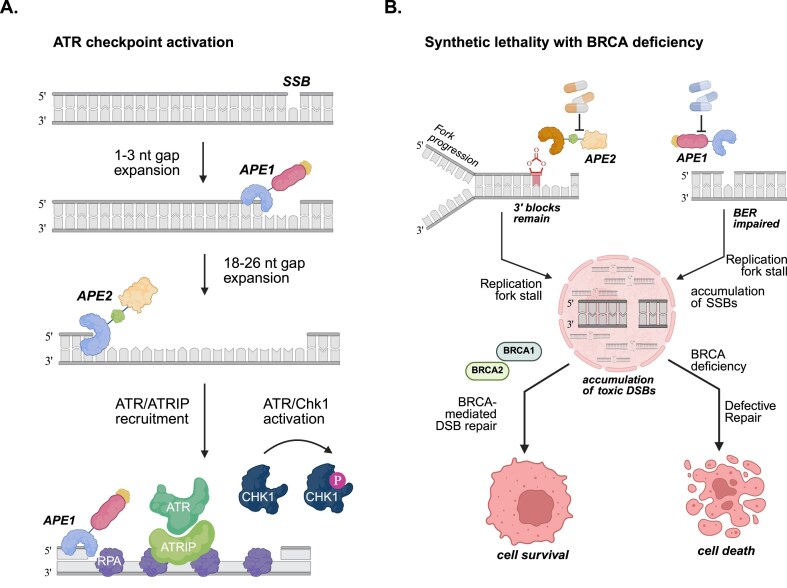
Coordinated role of APE1 and APE2 in DDR and synthetic lethality. (**A**) Mechanistic steps of APE1 and APE2 in the activation of ATR/Chk1 DDR pathway. (**B**) Synthetic lethality induced by inhibiting APE1 or APE2 in BRCA-deficient cancers. Created in BioRender. Grimsley, H. (2025; https://BioRender.com/sa9zn9k).

Step 1—APE1 initiates resection and recruits ATRIP: In Xenopus egg extracts, APE1 acts first at an SSB, initiating 3′–5′ end resection with its exonuclease activity (Fig. 4A). This generates short ssDNA gaps which RPA quickly coats, marking the site as replication-stress damage [48]. Depletion of APE1 abolishes ATR-Chk1 activation and prevents APE2 recruitment at SSB sites [48]. A follow up study in Xenopus extracts showed that the N-terminal motif of APE1 directly interacts with ATRIP, and recruiting it to ssDNA gaps, and activating ATR signaling in an RPA dependent or independent manner [122]. These findings indicate APE1 has a dual role: it both processes the break and serves as a scaffold to recruit ATR–ATRIP.
Step 2—APE2 extends resection and amplifies ATR Activation: In Xenopus extracts, APE2 is recruited to the ssDNA gaps and extends the resection initiated by APE1. APE2’s exonuclease activity, enhanced by PCNA, creates longer ssDNA gaps (∼18–26 nt), which allow robust RPA coating and full ATR checkpoint assembly as shown in pancreatic cancer cells [101] (Fig. 4A). APE2 also directly interacts with Chk1 to facilitate its phosphorylation by ATR. The recruitment of APE2 depends on its PCNA-binding PIP motif and ssDNA-binding Zf-GRF motif—domains essential for its SSB repair and checkpoint functions [99]. Mutating the Zf-GRF domain (which disrupts ssDNA/PCNA binding) abolishes APE2-driven RPA recruitment and ATR–Chk1 signaling, showing that both PCNA interaction and ssDNA binding are essential to APE2’s checkpoint role [101]. Thus, APE2 amplifies APE1’s initial damage response into a robust ATR–Chk1 checkpoint signal.

In summary, APE1 and APE2 function in a sequential two-step model: APE1 trims the 3′ end of the break first, and APE2 then amplifies the ssDNA signal to engage ATR/ATRIP. Neither enzyme can fully substitute for the other in checkpoint activation [122]. This two-step APE1–APE2 mechanism was first shown in the Xenopus laevis egg extract system. Parallel studies in human pancreatic cancer cells showed that the loss of APE2 significantly impairs ATR–Chk1 activation across diverse genotoxic stresses [101].

Beyond catalysis, APE1 also forms nucleolar condensates that concentrate ATR, TOPBP1, and ETAA1, supporting checkpoint activation independent of exogenous damage. [123]. The nonenzymatic activity of APE1 is mediated by the N-terminal NT33 motif, which is required for interactions with ATR, ATRIP, and RPA, highlighting a structural role for APE1 in DDR regulation [123]. These cellular studies indicate that the APE1/APE2-dependent ATR checkpoint activation mechanism is conserved in mammals. APE1 and APE2 appear to act sequentially in DDR, but how this coordination is regulated remains unclear. How is APE2 activated or regulated after APE1’s incision? It is not yet clear whether specific post-translational modifications or chromatin context dictate this coordination. A better mechanistic mapping could reveal new targets for tweaking the checkpoint in cancer cells.

## Cancer etiology

### Clinical implications of APE1 and APE2

Cancer cells experience high levels of replication stress and oxidative DNA damage, producing abundant AP sites that can block replication. APE1 is often upregulated in tumors, and this elevation may help cells cope with the high load of abasic sites and genotoxic stress, enabling cancer cells to better withstand chemotherapy or radiation [124]. Tumor tissues typically show elevated catalytically active, acetylated APE1 compared with normal tissues, reflecting enhanced repair capacity [125]. As a multifunctional protein, APE1 contributes to cancer progression through both its enzymatic DNA repair activity and its redox-regulatory effects on gene expression. For instance, in pancreatic cancer models, pharmacological blockade of APE1’s redox activity by E3330 treatment decreased NF-κB, AP-1 and HIF1α signaling and induced marked tumor growth inhibition *in vivo* [126]. Likewise, treatment with the endonuclease inhibitor—APE1 Inhibitor III in esophageal adenocarcinoma (EAC) model showed retardation in cancer cell growth *in vitro* and *in vivo* [127]. Together, these studies provide direct evidence that both redox signaling and endonuclease activity of APE1 are required to sustain tumor cell growth.

Clinically, high APE1 expression has been documented in prostate, lung, ovarian, colorectal, pancreatic, melanoma, head and neck, and other cancers, and generally correlates with advanced stage, chemoresistance, and poor survival, yet the underlying causes of APE1 overexpression in tumors remain unclear [124, 128–138]. For example, platinum-based chemotherapy induces APE1 in nonsmall cell lung cancer (NSCLC), where elevated levels predict invasion and shorter survival [139, 140]. Elevated APE1 levels have been shown to confer resistance to 5-fluorouracil in colon cancer, and alkylating agents in glioma [141–143]. Ovarian cancer displays increased APE1 protein expression and has been associated with poor survival and tumor progression [132, 144]. In EAC and multiple cancer cell line models, elevated APE1 levels dysregulate HR and the cell cycle, contributing to genome instability, tumorigenesis and chemoresistance [127]. Beyond DNA repair, the redox function of APE1 has a significant impact oncogenic pathways. The target genes under APE1 redox control include HIF-1α, p53, NF-κB, AP-1, and others, which are involved in hypoxic adaptation, cell cycle arrest, inflammation, and apoptosis evasion [67, 68, 70]. For instance, APE1-mediated reduction of HIF-1α enhances hypoxia-responsive transcription (e.g. VEGF expression), promoting tumor angiogenesis [68, 145]. Moreover, APE1 is detectable in serum (sAPE1), and elevated levels correlate with metastasis and poor prognosis, highlighting its potential as a noninvasive biomarker [146]. High serum APE1 levels have been reported in patients with lung, gastric, and other cancers.

APE2 is also frequently dysregulated in cancer. One study reported APE2 genomic alterations in ∼17% of tumors and elevated APE2 mRNA in multiple tumor types (kidney, breast, lung, liver, uterine) versus normal tissues. [13]. Other analyses found high APE2 mRNA/protein in hepatocellular carcinoma, prostate and pancreatic cancers, and multiple myeloma [13, 147]. In LIHC, APE2 upregulation correlates with higher Cyclin B1 and MYC expression, supporting cell-cycle progression and proliferation [148]. Functionally, APE2 promotes error-prone repair and replication stress tolerance, sustaining tumor growth. Paradoxically, in some cancers (lung, bladder, ovarian, cervical), higher APE2 expression correlates with better survival, suggesting its oncogenic impact may be tissue-specific [17].

Together, these observations emphasize the dual clinical impact of APE1 and APE2. High expression often signals enhanced DNA repair capacity, which can reduce the effectiveness of DNA-damaging therapies. At the same time, their divergent roles—in BER and redox signaling (APE1) versus replication stress response (APE2)—highlight opportunities for biomarker-driven prognosis and therapeutic targeting. Importantly, cancer risk linked to these enzymes may also reflect their roles in immune regulation, not solely DNA repair, underscoring the multifaceted implications of APE1 and APE2 dysregulation in oncology.

### Synthetic lethality in BRCA-deficiency

BRCA1 and BRCA2 are essential for high-fidelity repair of DSBs by the HR pathway [149]. Loss of BRCA1 or BRCA2 forces cells to rely on error-prone repair, producing genomic instability—a phenotype termed “BRCAness” [150]. Such tumors are hypersensitive to DNA-damaging agents, as seen with PARP inhibitors [151]. Large-scale CRISPR screens in BRCA1/2-deficient cells identified APE1 and APE2 as top synthetic lethal partners [15, 16, 47, 152].

Loss or inhibition of APE1 in HR-deficient cells leads to accumulation of abasic sites, which stall replication forks and collapse into DSBs. (Fig. 4B). In HR-proficient cells, these lesions are repaired, but in HR-deficient cells they are lethal. Consistently, small-molecule APE1 inhibitors display synthetic lethality in BRCA- and ATM-deficient models. [14]. Likewise, without APE2, BRCA-mutant cells cannot properly clean up blocked 3′ DNA ends at stalled forks, resulting catastrophic fork collapse. APE2 resects the 3′-blocked SSB ends, generating RPA-coated ssDNA tracts that activate the ATR/Chk1 checkpoint [17]. In HR-proficient cells, ATR/Chk1 can partly stabilize these forks and initiate alternative repair. However, in HR-deficient cells, APE2 deficiency leads to accumulation of 3′-blocking lesions such as unrepaired ribonucleotides which are lethal if unprocessed [16] In the absence of functional HR (e.g. BRCA mutations), these unresolved lesions from APE1 or APE2 loss become catastrophic, explaining the synthetic lethal relationship (Fig. 4B).

The synthetic lethal relationships of APE1 or APE2 with BRCA1/2 deficiencies establish them as promising drug targets, extending the PARP inhibitor therapeutic paradigm. Importantly, the lethal mechanisms differ: APE1 loss triggers unrepaired AP site accumulation, while APE2 loss prevents clearance of complex 3′ termini. Dissecting these distinct vulnerabilities is crucial to refine therapy design and minimize toxicity.

## Therapeutic targeting of APE1 and APE2

Given their essential roles in genome maintenance and cancer adaptation, APE1 and APE2 have emerged as promising therapeutic targets, with APE1 inhibitors already developed in the past two decades and APE2 representing a novel frontier for precision oncology. This section discusses key APE1 endonuclease and redox inhibitors and explores APE2 as a new therapeutic target.

### Targeting the endonuclease activity of APE1

The APE1 endonuclease activity is crucial for the BER pathway to eliminate AP sites. Inhibiting APE1’s repair function causes abasic sites to accumulate, resulting in chromosome aberrations (e.g. mitotic defects, telomere dysfunction) and genomic instability [93, 153]. APE1 inhibition is catastrophic in cells with deficiency in DNA repair pathways. Blocking APE1 in BRCA1-, BRCA2-, or ATM-deficient cells triggers synthetic lethality [14].

Several small-molecule APE1 endonuclease inhibitors have been explored (Table 1). Methoxyamine (MX), an alkoxyamine, was one of the first BER inhibitors targeting APE1 indirectly [154]. MX reacts with the open-ring form of an AP site to form a stable imine adduct, thereby blocking APE1’s incision. In preclinical models, MX potentiates alkylator chemotherapy such as temozolomide (TMZ), methyl methanesulfonate (MMS) by forcing AP site accumulation [155, 156]. A clinical formulation of methoxyamine (TRC102) did enter clinical trials. TRC102 (a clinical formulation of MX) advanced into Phase I trials: in combination with TMZ it increased tumor DNA damage and produced some partial tumor responses in refractory solid cancers (NCT01851369) [157]. TRC102 has also been evaluated in combination with platinum-based chemotherapy and radiation in NSCLC (NCT05198830), aiming to overcome resistance by targeting BER repair of treatment-induced lesions [158]. However, MX-based strategies have not yet clearly improved clinical outcomes.

**Table 1.** List of APE1 endonuclease inhibitors

Inhibitor name	Mechanism of action	Clinical implications
**CRT0044876** (endonuclease)	Binds to APE1’s active site, inhibiting its AP endonuclease activity	Preclinical studies show synergy with alkylating agents (e.g. temozolomide).
**Methoxyamine** (endonuclease)	Indirect inhibitor. Blocks APE1 endonuclease activity by modifying AP sites.	Clinical studies in combination with alkylating agents and radiation therapy
**Lucanthone** (endonuclease)	Noncompetitive inhibitor. Binds to APE1’s hydrophobic site, altering the active site and disrupting endonuclease activity.	Clinical studies as an adjuvant for tumour radiotherapy.
**Myricetin** (endonuclease)	Endonuclease function inhibited	Preclinical studies demonstrate significant anticancer activity across multiple tumour types.
**AR03** (endonuclease and redox)	Inhibits both endonuclease and exonuclease activity	Preclinical studies show AR03 to potentiate the cytotoxicity of DNA damaging agents, such as methyl methanesulfonate and temozolomide.
**APE inhibitor III** (endonuclease)	Competitive inhibitor that blocks APE1’s active site inhibiting endonuclease activity	Preclinical studies show synergistic effects when combined with other agents such as talazoparib.

Lucanthone (Miracil D), an old antiparasitic agent, was later found to inhibit APE1’s endonuclease activity and sensitize cells to TMZ and MMS, and structural studies suggest it binds APE1’s hydrophobic pocket [159, 160]. However, lucanthone’s specificity is questionable since it also intercalates DNA and inhibits topoisomerase II [161].

High-throughput screening have identified more selective hits. For example, the indole derivative CRT0044876 (7-nitroindole-2-carboxylic acid) inhibits APE1 *in vitro* and sensitizes cells to MMS/TMZ, but it suffered from poor solubility and cell permeability [162, 163]. A fluorescence-based screen of 60 000 molecules identified several candidate APE1 inhibitors (AR01, AR02, AR03, AR06), with AR03 being the most potent. AR03 inhibits APE1 (both endonuclease and 3′–5′ exonuclease functions) at low micromolar range and increases chemosensitivity in cell culture, though it’s *in vivo* efficacy remains untested [164]. “APE inhibitor III” is a widely used commercially available endonuclease inhibitor of APE1 [165]. In one study, combining APE inhibitor III with the PARP inhibitor talazoparib and DNA methyltransferase inhibitor decitabine synergistically enhanced cytotoxicity in myeloid malignancies [166].

Some agents target both APE1’s repair and redox functions. Gossypol, a natural polyphenol, binds APE1 and block its DNA repair activity while also disrupting redox interactions [167]. In preclinical studies, gossypol synergizes with DNA-damaging drugs (cisplatin, MMS) to enhance cell death. A Phase II trial (NCT00540722) added gossypol to cisplatin/docetaxel in NSCLC patients with high APE1 tumors, but it did not significantly improve outcomes (though there was a nonsignificant trend toward longer survival) [168]. The gossypol derivative AT-101 also sensitized cancer cells to cisplatin by modulating the APE1/IL-6/STAT3 axis in NSCLC models [169], although direct APE1 binding by AT-101 has not been fully confirmed. *In vivo*, AT-101 impaired gastric cancer cell renewal and migration, increasing chemotherapy sensitivity [170]. These results suggest that dual inhibition of APE1 repair and redox functions may be a useful strategy, but better agents are needed.

A recent study identified a novel APE1 endonuclease inhibitor, no. 0449-0145, via *in silico* docking and AP-site cleavage assays [171]. This compound has potent antitumor effects in NSCLC models: it induces DNA damage and triggers inflammatory cell death pathways (apoptosis, pyroptosis, necroptosis), and it can overcome both cisplatin- and EGFR-inhibitor resistance [171]. Further biochemical studies are needed to confirm direct binding of no. 0449-0145 to APE1. Similarly, a recent fragment-based X-ray screen yielded bona fide APE1-binding ligands (XPTx-0091 and XPTx-0387) with sub-micromolar potency and sensitize cells to alkylation damage [172]. These advances show progress toward direct APE1 inhibitors.

### Targeting the redox function of APE1

The N-terminal region of APE1 controls the redox activity and regulates key TFs critical for tumor survival [59]. Several inhibitors of APE1’s redox function have been developed (Table 2), the most advanced being APX3330—a quinone derivative that drives the formation of disulfide bonds in APE1’s redox-active cysteines, thereby preventing APE1 from reducing its TF targets [173, 174]. APX3330 has demonstrated broad anticancer activity in preclinical models: it inhibits proliferation and migration of pancreatic and lung cancer cells, and it also suppresses tumor angiogenesis [175]. APX3330 significantly enhanced cisplatin-induced cytotoxicity in NSCLC cells and reduced their migratory/invasive capacity [176]. Co-treatment with APX3330 also improved the efficacy of docetaxel in triple-negative breast cancer models by inhibiting cell proliferation and invasion [177]. A Phase I trial (NCT03375086) in advanced solid tumor patients established APX3330’s safety, pharmacokinetics, and target engagement at tolerable oral doses [178]. Beyond oncology, APX3330 also succeeded in a Phase II trial for diabetic retinopathy (NCT04692688), significantly slowing disease progression versus placebo [179]. These findings highlight that APE1 inhibitor APX3330 may have broad clinical utility in diseases driven by oxidative stress and abnormal transcriptional signaling, not only in cancer.

**Table 2.** List of APE1 redox inhibitors

Inhibitor name	Mechanism of action	Clinical implications
**Curcumin** (redox)	Direct inhibitor of APE1’s redox function.	Early-phase clinical trials have a role in chemoprevention and therapy.
**Resveratrol** (redox)	Indirect inhibition of APE1’s redox function.	Well tolerated but has poor bioavailability.
**ML199** (redox)	Selective inhibition of APE1 endonuclease activity.	Enhances cytotoxicity of DNA-damaging agents in preclinical models.
**APX3330** (E3330) (redox)	Target redox function (Ref-1 domain), blocking TF activation (e.g. HIF-1α, NF-κB).	Phase I/II trials for pancreatic, colorectal, and blood cancers; reduces angiogenesis and tumour growth.
**APX2009** (redox)	Selectively inhibits APE1’s redox function.	Pre-clinically demonstrates reduction in proliferation, migration and invasion of cancer cells. Advanced to Phase I and II trials. Positioned as the next-generation candidate.
**Gosssypol (AT-101)** (redox)	Direct binding of APE1 results in conformation change and inhibition of both endonuclease and redox function.	Early-phase clinical trials is shown to be well tolerated and combined with docetaxel and fluorouracil.
**Spiclomazine** (redox)	Inhibits APE1 and NPM1 interaction.	Pre-clinical studies show inhibition of the survival of pancreatic cancer cells harbouring Kras mutants.
**SB 206553** (redox)	Inhibits APE1 and NPM1 interaction.	Pre-clinical studies show sensitization of various cancer cell lines to DNA-damaging agents, including bleomycin.

Second-generation analogs (APX2007, APX2009, APX2014, etc.) have been developed with improved potency. For example, APX2009 retains anti-tumor activity but also protects normal neurons from cisplatin-induced toxicity [173]. Several natural compounds also inhibit APE1 redox activity: curcumin has been reported to suppress APE1’s redox activation of AP-1 and NF-κB, thereby reducing expression of TF targets [180]. Another promising concept is to combine APE1-redox inhibition with immunotherapy. APE1-driven redox signaling elevates immunosuppressive cytokines (IL-6, TGF-β) and increases PD-L1 expression via NF-κB/STAT3. Indeed, co-expression of APE1 and PD-L1 in tumors correlates with aggressive disease and predicts poor response to checkpoint blockade [181]. Thus, blocking APE1 may reprogram the tumor immune microenvironment. Moreover, some APE1 inhibitors trigger pyroptosis and necroptosis, which release neoantigens and could further prime anti-tumor immunity [171]. Together with DNA repair targeting, APE1-redox blockade opens a new avenue to exploit cancer vulnerabilities in signaling and immune evasion.

### Emerging efforts to target APE2

Unlike APE1, APE2 currently has no approved inhibitors, but its critical role in replication-stress repair makes it an attractive novel target. APE2 loss is synthetically lethal in HR-deficient cancers [16, 47]. Since PARP-inhibitor–resistant tumors often restore HR or upregulate backup end-joining pathways, adding an APE2 inhibitor could overcome this resistance. Similarly, combining an APE2 inhibitor with ATR or CHK1 inhibitors might synergistically suppress the replication stress response, since APE2 acts upstream of ATR–Chk1 [101]. Preclinical BRCA1/2-deficient tumor models (PDX or GEMMs) should be used to test whether APE2 inhibitors can shrink tumors or synergize with PARPi/chemotherapy *in vivo*.

Recently, one study reported the first APE2 inhibitor: Celastrol, a plant-derived triterpene. In preclinical studies, celastrol binds to APE2 and blocks its ssDNA binding and exonuclease functions, thereby impairing ATR–Chk1 signaling in cancer cells [101]. Functionally, celastrol treatment (or APE2 knockdown) strongly sensitizes tumor cells to DNA-damaging chemotherapy. For instance, in pancreatic cancer models celastrol dramatically enhances cisplatin killing. [101]. Celastrol is already in early phase clinical trials (NCT05494112, NCT05413226) for safety evaluation [182, 183], highlighting its potential to be developed as a cancer therapeutic.

Overall, targeting APE2 holds promise in several ways:

- Monotherapy in HR-deficient tumors: In BRCA1/2- or related repair-deficient cancers, APE2 loss is synthetically lethal, so an APE2 drug could be effective on its own.
- Combination with other targeted agents: An APE2 inhibitor might deepen responses to PARP inhibitors, or synergize with ATR/CHK1 inhibitors, to overcome resistance.
- Combination with DNA-damaging therapy: Because APE2 repairs replication-associated damage, blocking it should make tumors more sensitive to chemotherapies or radiation.

APE1 and APE2 are structurally similar in their catalytic cores, the current APE1 inhibitors can potentially cross-react with APE2. In fact, many APE1-active site inhibitors likely also bind APE2’s catalytic pocket, analogous to early PARP inhibitors that hit both PARP1 and PARP2 [184, 185]. This challenge underscores the need for isoform-selective drug design—future inhibitors should exploit structural differences outside the conserved active site (such as APE2’s unique zinc-finger or PCNA-binding motif) to achieve specificity. Addressing this issue in drug development will require exploiting subtle structural differences between APE1 and APE2 without unintended inhibition of the other, thereby minimizing off-target effects.

## Discussion

### Key insights and implications

APE1 and APE2 are central guardians of genome stability, but their roles are mechanistically distinct. APE1 is the dominant AP endonuclease, with an added redox regulatory function that modulates TFs. In contrast, APE2 exhibits weak intrinsic AP endonuclease and exonuclease activity (several orders of magnitude lower than APE1) but acquires functional significance through its PIP motif and Zf–GRF domain tether APE2 to replication forks when bound to PCNA, stimulating its processivity during replication stress. Thus, APE1 operates as a catalytically dominant repair enzyme, while APE2 acts as a PCNA-dependent signaling nuclease specialized for replication-associated responses.

Clinically, both enzymes are frequently upregulated in tumors, likely reflecting adaptation to the heightened replication stress and oxidative burden of cancer metabolism. APE1 promotes tumor progression via dual mechanisms: clearing abasic sites to sustain viability and activating redox-dependent transcriptional programs that drive angiogenesis, proliferation, and immune evasion. Although less studied, APE2 upregulation has been linked to poor prognosis in hepatocellular carcinoma and hematologic cancers, with pan-cancer analyses showing elevated mRNA in kidney, lung, and breast tumors. These patterns suggest APE2 could serve as a biomarker and therapeutic vulnerability.

The concept of synthetic lethality underscores their therapeutic relevance. Recent CRISPR screens confirm that BRCA1/2-mutant cells are dependent on APE2, and that APE2 nuclease-dead mutants fail to rescue viability. In HR-deficient cancers, loss of APE1 leads to toxic accumulation of AP sites at replication forks, while loss of APE2 prevents processing of blocked 3′ termini and ATR signaling, causing fork collapse. These findings establish APE1 and especially APE2 as attractive synthetic-lethal targets and prompting the development of selective small-molecule inhibitors.

### Current knowledge gaps

Despite these advances, important questions remain. How do cells prioritize APE1 versus APE2 at overlapping substrates? To what extent is APE2 redundant with or complementary to APE1 in immunoglobulin diversification (SHM/CSR), where conflicting results persist? The upstream signals that route replication stress responses through APE2-dependent ATR activation versus other pathways also remain undefined. At the translational level, can APE1 and APE2 expression levels stratify patients for DDR-targeted therapies, much as BRCA status predicts PARP inhibitor response? Finally, what structural differences outside the conserved active site can be exploited for isoform-selective therapeutic targeting?

### Therapeutic opportunities

Therapeutically, several inhibitors are under exploration. For APE1, the redox function inhibitor APX3330 has advanced to phase II trials, demonstrating biological activity with acceptable tolerability. Next-generation APX analogs improve on APX3330’s potency and pharmacokinetics. In addition to APX compounds, fragment-based and virtual screening have yielded novel direct-binding APE1 inhibitors with sub-micromolar activity.

In contrast, APE2-targeted therapies are still nascent. Celastrol was recently identified as the first small-molecule APE2 inhibitor and it impairs APE2’s ssDNA binding and 3′–5′ exonuclease activity, attenuating ATR-Chk1 signaling. APE2 appears to be a selective lethal target in BRCA/HR-deficient tumors. Combining APE2 inhibitors with PARP inhibitors or ATR/CHK1 inhibitors is also attractive, since APE2 lies upstream of ATR (dual blockade could collapse the replication-stress response). Preclinical models (e.g. BRCA-mutant xenografts) should be used to test these combinations and to identify biomarkers of response.

A key consideration in all these efforts is specificity. APE1 and APE2 share nearly identical catalytic cores. Therefore, drug discovery must exploit divergent structural features to achieve isoform-selectivity.

### Future directions

Future research on APE1 and APE2 should focus on closing critical mechanistic and translational gaps:

- Mechanistic dissection: High-resolution structural studies of full-length APE2 (currently lacking) are needed to reveal how its unique domains—including the PIP motif and Zf–GRF motif—coordinate with the nuclease core to regulate substrate engagement and processivity in coordination with PCNA.
- Therapeutic development:
  - Generation of specific inhibitors: Structure-guided drug discovery should prioritize the development of isoform-selective inhibitors that exploit divergent structural elements of APE1 and APE2, beyond their conserved catalytic pockets.
  - Testing of current inhibitors: Existing APE1 inhibitors (e.g. CRT0044876, no. 0449-0145, APX3330) should be systematically evaluated for potential off-target effects on APE2. Such testing will help refine specificity, reduce toxicity, and ensure more precise therapeutic targeting.
- Synthetic lethality exploitation: Preclinical *in vivo* models of BRCA1/2-deficient cancers are essential to validate APE1/APE2 inhibition as a therapeutic window, define biomarkers of sensitivity, and identify resistance mechanisms. Combining APE inhibitors with PARP, ATR, or Chk1 inhibitors could extend the PARP inhibitor paradigm.
- Biomarker discovery: Large-scale clinical datasets should be mined to correlate APE1/APE2 expression and mutational status with patient outcomes, potentially informing patient stratification and guiding combination therapies with DDR-targeted agents.
- Role of APE2 in mammalian DNA demethylation: One such area is the potential role of APE2 in active DNA demethylation, based on findings in *Arabidopsis thaliana*. Active demethylation of 5-methylcytosine in DNA involves a BER-like mechanism [186]. Currently, the role of APE2 in active DNA demethylation is evident in plant models [187]; however, its role in mammalian DNA methylation remains speculative. Future studies should explore whether APE2 participates in cytosine demethylation in human cells.

In summary, defining the structures, functions, and selective vulnerabilities of APE1 and APE2 will pave the way for precision oncology strategies that exploit their synthetic-lethal interactions and therapeutic potential.

## Data Availability

No new data were generated or analysed in support of this research.
